# Robotic-assisted total knee arthroplasty demonstrates superior early functional outcomes compared with conventional total knee arthroplasty: a quasi-randomised controlled trial

**DOI:** 10.1186/s10195-026-00979-7

**Published:** 2026-11-03

**Authors:** Filippo Migliorini, Luise Schäfer, Andrea Nobili, Daniel Kämmer, Jens Schneider, Andreas Bell

**Affiliations:** 1 Department of Orthopaedic and Trauma Surgery, Eifelklinik St. Brigida, 52152 Simmerath, Germany; 2 Department of Trauma and Reconstructive Surgery, University Hospital of Halle, Martin-Luther University Halle-Wittenberg, Ernst-Grube-Street 40(Saale), 06097 Halle, Germany; 3 Department of Orthopaedic and Trauma Surgery, Academic Hospital of Bolzano (SABES-ASDAA), 39100 Bolzano, Italy; 4 Department of Life Sciences, Health, and Health Professions, Link Campus University, Via del, 00165 Rome, Italy

**Keywords:** Computer-assisted, Knee osteoarthritis, Replacement, VAS, Patient satisfaction, Orthopaedic surgery, Patient-reported outcomes

## Abstract

**Background:**

Robotic-assisted total knee arthroplasty (TKA) has gained popularity over the last decade, with claimed advantages in implant positioning accuracy and soft-tissue balance. Whether these technical advantages translate into better clinical outcomes for patients remains debated. This investigation compared 1-year patient-reported outcomes between robotic-assisted and conventional TKA performed within the same institution but following structurally different surgical workflows.

**Methods:**

This quasi-randomised controlled trial enrolled patients who underwent primary TKA at the Department of Orthopaedic and Trauam Surgery of the Eifelklink St. Brigida (Simmerath, Germany) between 2022 and 2025. Group allocation was determined by surgeon assignment through the institutional digital scheduling system. All patients received the same implant (Smith & Nephew Legion Genesis II, posterior-stabilised). Robotic-assisted procedures were performed using the CORI Robotic System (Smith & Nephew, UK) following a four-dimensional personalised alignment workflow (coronal, sagittal, transversal and balancing) guided intraoperatively by the Knee Balancing Score (KBS). Conventional procedures followed a femur-first technique with adjusted mechanical alignment. Outcomes included WOMAC total and subscores, visual analogue scale (VAS), Tegner activity score and a dedicated functional questionnaire, all assessed at 1-year follow-up.

**Results:**

A total of 845 patients were included (robotic-assisted: *n* = 390; conventional: *n* = 455). At 1-year, 383 and 445 patients completed follow-up, respectively. The two groups were comparable at baseline. The robotic-assisted cohort showed significantly better results across WOMAC and related subscales (*P* < 0.001), VAS (*P* < 0.001), Tegner (*P* < 0.001), functional and satisfaction items, including walking distance and knee stability. No difference was found in the use of walking aids.

**Conclusions:**

Robotic-assisted TKA was associated with superior functional and patient-reported outcomes at the 1-year follow-up. The clinical benefit observed is likely not attributable to robotic precision alone, but reflects the structured, personalised intraoperative workflow that robotic assistance enables.

*Trial Registry* German Registry of Clinical Trials (ID DRKS00030614).

## Introduction

Total knee arthroplasty (TKA) is among the most frequently performed orthopaedic procedures worldwide and, in most patients, provides reliable pain relief and functional improvement [1–3]. Yet a non-negligible proportion of patients, estimated at around 15–20% in most series, remain dissatisfied after surgery, despite what appears to be a technically good result [4, 5]. Over the past two decades this paradox has driven substantial rethinking about how TKA should be planned and executed [6, 7]. The traditional mechanical alignment paradigm, which targets a neutral hip–knee–ankle axis for every patient regardless of individual anatomy, has been progressively challenged [8–10]. Population studies show that roughly half of non-arthritic individuals lack a neutral mechanical axis [11, 12], and that forcing all knees into a fixed neutral position may distort the native soft-tissue envelope rather than restore it [13, 14]. This observation has led to more individualised approaches, from kinematic alignment to restricted kinematic alignment and, more recently, functional alignment, a philosophy that aims to reconstruct constitutional knee anatomy within defined safe boundaries while simultaneously achieving a measurable soft-tissue balance [15–17]. Robotic-assisted TKA has emerged as a technology capable of supporting these more complex alignment strategies [18, 19]. Unlike conventional instrumentation, robotic platforms allow three-dimensional pre-operative planning, real-time intraoperative feedback on implant position, and objective quantification of mediolateral compartment gaps before any bone is resected [20, 21]. These capabilities are particularly relevant when the surgical goal goes beyond simply cutting at a fixed angle and instead involves integrating multiple anatomical variables simultaneously [22, 23]. Despite this, randomised evidence comparing robotic and conventional TKA has yielded heterogeneous results for patient-reported outcomes [24–26]. However, most studies use the robotic device as the only variable. Certainly, robotic-assisted and conventional surgery often involve not just different tools but different planning philosophies and intraoperative decision-making frameworks [27, 28]. Comparing outcomes without acknowledging this difference risks attributing to the robot what is, in fact, the effect of the workflow.

The present quasi-randomised controlled trial was designed with this philosophy in mind. We compared 1-year patient-reported outcomes between robotic-assisted TKA, performed using a four-dimensional personalised alignment workflow with intraoperative balancing guided by the Knee Balancing Score (KBS) [29], and conventional freehand TKA performed using a femur-first technique with adjusted mechanical alignment. Our hypothesis was that the structured, reproducible workflow enabled by robotic assistance would result in meaningfully better functional outcomes for the patient at the 1-year follow-up.

## Methods

### Study design

The present quasi-randomised controlled trial followed the 2025 CONsolidated Standards of Reporting Trials (CONSORT) statement and established standards for reporting clinical research [30, 31]. All patients presenting for pre-operative assessment before primary TKA at the Department of Orthopaedic Surgery of the Eifelklinik St. Brigida in Simmearth (Geramny) from 2022 to 2025 were prospectively invited to participate in this investigation. This study was conducted in accordance with the principles of the Declaration of Helsinki, its subsequent amendments and general recommendations [31]. Ethics approval was granted by the Medical Council of Düsseldorf, Germany. The protocol of the present study has been previously published. The study was prospectively registered in the **. The institution where the surgeries were performed is accredited by ‘EndoCert’ (EndoCert certificate, Centres of German Endoprosthetics, German Society for Orthopaedics and Traumatology) [32], which supervises and certifies the quality of the surgical procedures. All procedures were performed by EndoCert-certified senior arthroplasty surgeons who were well beyond their learning curve for both conventional and robotic-assisted TKA, with more than 1500 TKAs each.

### Eligibility criteria

The inclusion criteria included: being older than 18 years; being able to provide informed consent; and having symptomatic knee osteoarthritis graded stage II to IV using the Kellgren–Lawrence classification [33]. The exclusion criteria consisted of: the presence of acute or chronic inflammatory diseases, cancer, pregnancy or breastfeeding, uncontrolled clotting disorders, abnormal blood cell counts, severe peripheral neuropathy, vascular diseases, peripheral ulcers, missing data related to the study’s endpoints, blood tests performed on days other than post-operative day (POD) 1 or 5, an inability to comply with the post-operative management protocol, or any other condition that could influence study outcomes.

### Randomisation

Patients who contacted the ** for consultation were automatically assigned to the first available outpatient appointment via the institution digital scheduling system. Appointment allocation was computer-generated based on outpatient availability. Two senior arthroplasty surgeons routinely performed robotic-assisted TKA, whereas two senior arthroplasty surgeons routinely performed conventional TKA. The administrative personnel who answered telephone calls and managed appointments were not informed of the present study.

### Surgical technique

All patients underwent identical clinical, imaging and anaesthesia protocols before and after surgery. Each patient received a single intravenous dose of 1.5 g cefuroxime at anaesthesia induction, and pain was managed with a continuous femoral nerve block maintained for 48 h. Every surgery was performed using the same standard medial parapatellar approach. All robotic-assisted surgeries were performed using the CORI Robotic System (Smith & Nephew, UK). All implant components (Smith & Nephew Legion Genesis II, posterior-stabilised, polyethylene liner) were placed according to the manufacturer’s guidelines. Palacos cement was used for both femoral and tibial fixation. At the end of surgery, 1 g of tranexamic acid was injected intra-articularly. Enoxaparin sodium (40 mg/0.4 mL) was initiated 12 h post-operatively and administered daily for 6 weeks as anti-thrombotic prophylaxis. Standardised physiotherapy protocols were followed, with physiotherapists providing care starting from the first POD. The minimum hospital stay was 5 days; it was extended if patients experienced post-operative setbacks, such as reduced knee mobility, difficulty walking or trouble with physiotherapy exercises. Starting on the second POD, patients participated in two daily physiotherapy sessions, each lasting 60 min, of continuous passive motion to increase knee flexion and extension, with the therapist progressively improving their range of motion (ROM). Discharge was based on achieving approximately 90° of flexion. Ambulation began under supervision from POD-2, and stair use started on POD-4. Each patient then received a tailored rehabilitation program lasting at least 3 weeks, either as an inpatient or an outpatient. Any deviation from the planned surgical or rehabilitation approach resulted in exclusion from the study.

### Robotic-assisted group: alignment philosophy and surgical workflow

All robotic-assisted procedures were performed following a structured four-dimensional intraoperative workflow, integrating personalised alignment decisions across four sequential domains: coronal plane, sagittal plane, transversal plane and soft-tissue balancing.

Pre-operative planning was based on long-leg radiographs with measurement of the medial proximal tibial angle (mPTA), lateral distal femoral angle (LDFA), arithmetic hip–knee–ankle angle (aHKA = mPTA − LDFA) and joint line obliquity (JLO = mPTA + LDFA) according to the Coronal Plane Alignment of the Knee (CPAK) classification [34]. Three morphotype-specific intervention categories were defined: Type A (aHKA ≤ ±2°), with reconstruction of both mPTA and LDFA guided by articular surface references; type B (aHKA ±2.5° to ±5°), with mPTA reconstructed to a maximum of 86–90° and LDFA to a maximum of 87–92°; and type C (aHKA > ± 5°), with strict adherence to the same mPTA and LDFA limits and soft-tissue release or higher constraint level planned as exit strategy if balance targets could not be achieved.

In the coronal plane, the femoral component was positioned to match the native LDFA, with a final hip–knee–ankle (HKA) target of 177°–182°. In the sagittal plane, femoral component size was selected using posterior referencing to match the posterior condyle offset to avoid anterior cortex notching, mediolateral overhang and patellofemoral overstuffing, with femoral flexion set between 0° and 6° and tibial slope limited to 3°; combined femoro-tibial flexion was kept below 10°. In the transversal plane, femoral rotation was referenced to the patient’s native posterior condylar axis and posterior femoral condylar morphology, within a range of 1° internal to 4° external rotation; tibial rotation was set using Akagi’s line.

Intraoperative soft-tissue balancing was examined prior to bone resection. After surgical exposure, systematic osteophyte removal and patellar displacement, the robotic system captured passive limb position in full extension and at 90° of flexion under standardised varus–valgus stress. Balance was classified using the Knee Balancing Score (KBS), based on the absolute mediolateral gap difference [29]. Target balance was KBS E1 or E2 in extension and F1 or F2 in flexion. When targets were not met, the virtual implant plan was modified (adjusting femoral coronal position, rotation, tibial varus/valgus or slope) before committing to bone cuts. The final plan was accepted when KBS targets, HKA range and joint line height within ±2 mm of the native were all satisfied simultaneously.

### Conventional group: alignment philosophy and surgical workflow

Conventional freehand procedures were performed using a restricted kinematic philosophy with a femur-first approach [35, 36]. The distal femoral cut was made perpendicular to the femoral mechanical axis, with the standard target of 0° of valgus/varus at the level of the distal femur. Femoral rotation was set using the posterior condylar axis with standard 3° of external rotation, cross-referenced with the transepicondylar axis and the anterior cortex. The proximal tibial cut was made perpendicular to the tibial mechanical axis with approximately 3° of posterior slope. Individual intraoperative adjustments to the component position could be made based on the surgeon’s clinical assessment, but without a systematic pre-operative morphotype-based plan, virtual gap simulation or quantitative soft-tissue balance measurement. Soft-tissue balance was assessed manually using tension gauges or spacer blocks, with releases performed when imbalance was clinically evident.

### Outcomes of interest

**Fig. 1:**
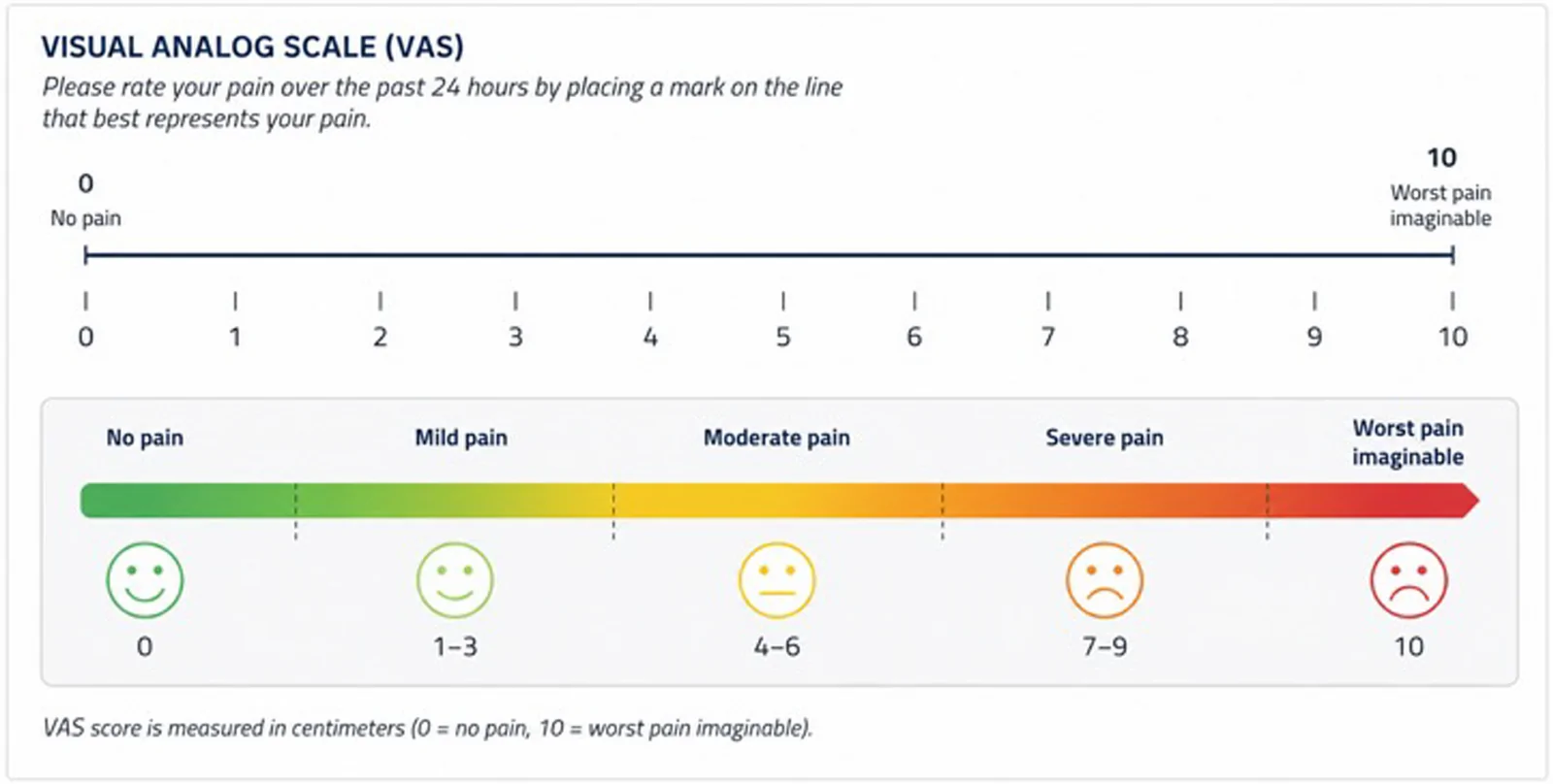
VAS

The outcomes of interest in the present study were pre-defined in the study protocol and included both baseline and 1-year post-operative patient-reported outcome measures (PROMs) and functional assessments following total knee arthroplasty. The evaluated PROMs included the Tegner activity score [37], visual analogue scale (VAS, Fig. 1) [38], WOMAC Pain, WOMAC Stiffness, WOMAC Function and WOMAC Total scores [39]. WOMAC subscores were calculated by summing the corresponding questionnaire items and the WOMAC Total score was obtained by summing all WOMAC domains.

**Fig. 2:**
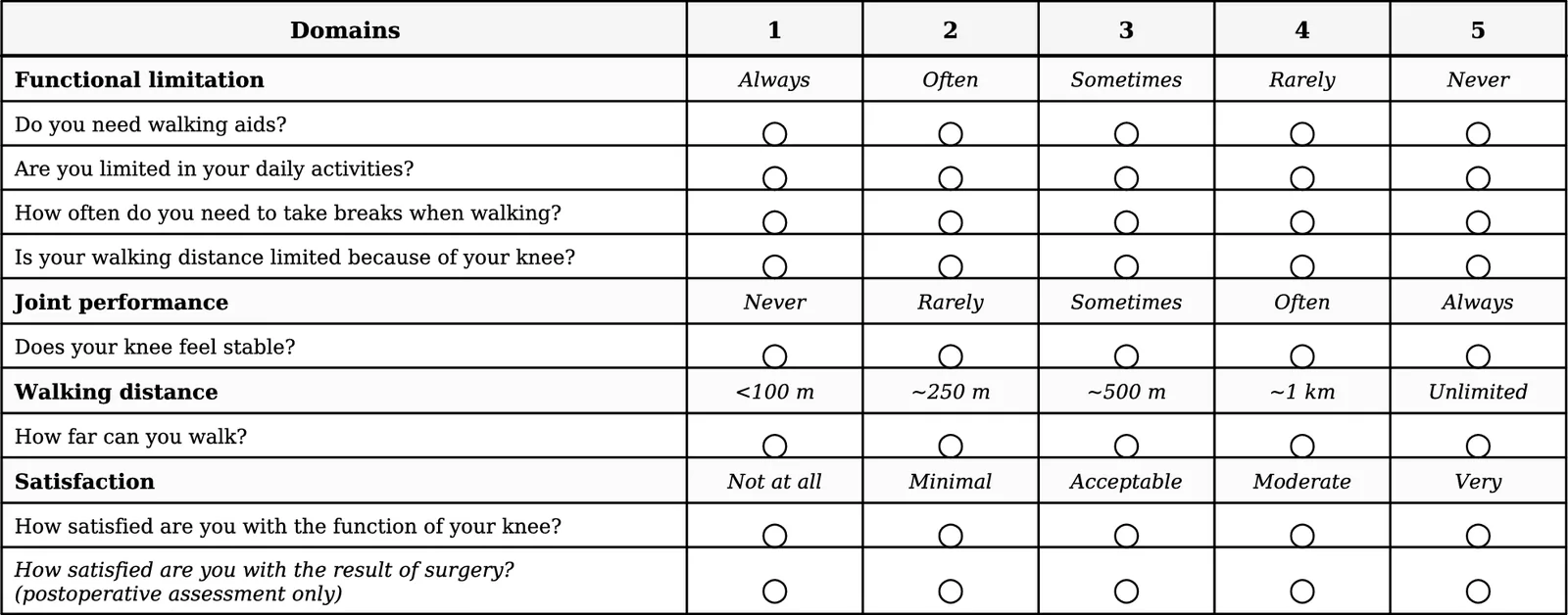
Assessment questionnaire

An additional questionnaire was administered to assess functional and satisfaction-related outcomes, which is routinely performed at our institution within the EndoCert quality-assurance framework (Fig. 2). This questionnaire was administered on admission and at the follow-up. At the 1-year follow-up, patient satisfaction with the overall outcome of the procedure was also evaluated. Baseline demographic variables, including age, body mass index (BMI), sex distribution and operated side, were also analysed to assess pre-operative comparability between the cohorts.

### Statistical analysis

The main author (**) performed the statistical analyses using IBM SPSS Statistics (version 25.0, IBM Corp., Armonk, NY, USA). Continuous variables were reported as mean and standard deviation, whereas categorical variables were expressed as absolute numbers and percentages. Baseline demographics, baseline PROMs and 1-year follow-up outcomes were compared between the robotic-assisted and conventional total knee arthroplasty cohorts. Continuous variables were analysed using the independent-samples *t*-test with Welch’s correction when appropriate. Mean differences (MDs) with corresponding 95% confidence intervals (95% CI) were calculated for all continuous outcomes. Categorical variables, including sex distribution and operated side, were analysed using the chi-squared test and reported as odds ratios (ORs) with 95% confidence intervals. Statistical significance was defined as *P* < 0.05.

## Results

### Patient enrolment

**Fig. 3:**
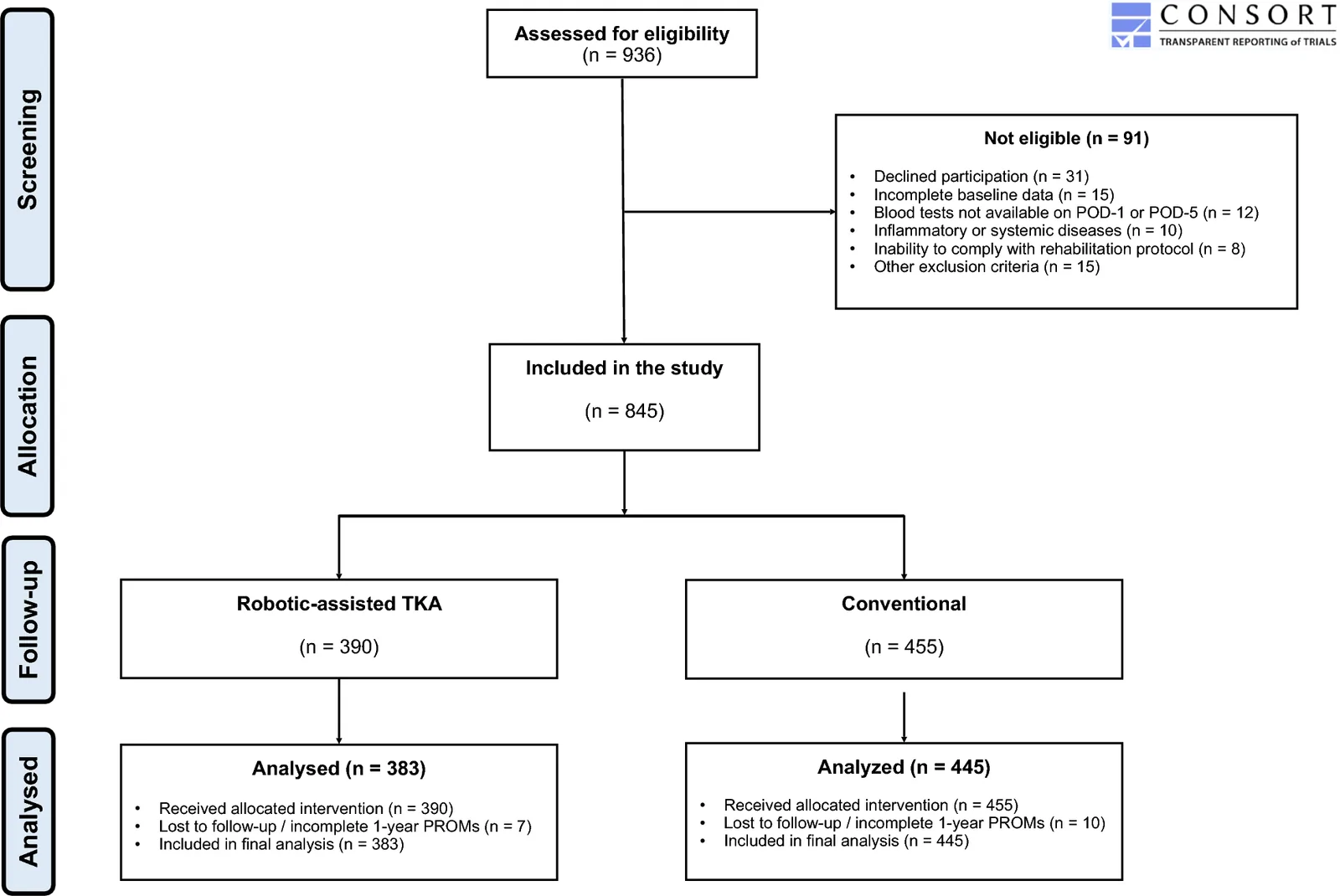
Flow chart of patient enrolment

A total of 936 patients were assessed for eligibility. Of them, 91 patients were excluded. Ultimately, 845 patients were included in the study. Among them, 390 patients were allocated to robotic-assisted TKA and 455 to the conventional TKA. At the 1-year follow-up, seven patients in the robotic-assisted cohort and ten patients in the conventional cohort had incomplete PROMs or were lost to follow-up. Some patients could not be reached by telephone despite repeated attempts, whereas others declined to complete the post-operative questionnaires due to time constraints, although they reported being satisfied with the surgical outcome. Therefore, 383 patients in the robotic-assisted cohort and 445 patients in the conventional cohort were included in the final analysis (Fig. 3).

### Patient demographics

A total of 828 patients were included in the final analysis. The robotic-assisted cohort comprised 383 patients, whereas the conventional cohort comprised 445 patients. The mean age was 67.4 ± 8.3 years in the robotic-assisted cohort and 67.0 ± 8.2 years in the conventional cohort. Mean BMI was 31.0 ± 5.7 and 30.2 ± 5.1, respectively. The robotic-assisted cohort comprised 219 women (57.2%), whereas the conventional cohort comprised 279 women (62.7%). Right-sided procedures were performed in 199 patients (52.0%) in the robotic-assisted cohort and in 229 patients (51.5%) in the conventional cohort. The two groups were comparable on admission (Table 1).

**Table 1:** Patient demographics (the effect size was evaluated as OR or MD for dichotomous or continuous variables, respectively)

Variable	Robotic-assisted (*N* = 383)	Conventional (*N* = 445)	*P*-value
Age	67.40 ± 8.34	67.03 ± 8.15	0.489
BMI	31.01 ± 5.69	30.23 ± 5.10	0.026
Women	219 (57.2%)	279 (62.7%)	0.122
Right side	199 (52.0%)	229 (51.5%)	0.816
Tegner	2.06 ± 1.71	1.95 ± 1.75	0.315
VAS	8.25 ± 1.01	8.19 ± 1.02	0.316
WOMAC pain	13.90 ± 3.86	13.96 ± 3.69	0.789
WOMAC stiffness	5.63 ± 2.06	5.73 ± 1.92	0.423
WOMAC function	47.30 ± 13.37	47.50 ± 13.19	0.794
WOMAC total	66.83 ± 16.99	67.19 ± 16.23	0.715
Walking aids	1.85 ± 1.23	2.01 ± 1.34	0.062
Restricted daily activities	4.01 ± 0.90	3.97 ± 0.92	0.505
Need for breaks when walking	3.75 ± 0.90	3.75 ± 0.92	0.979
Walking distance limitation	4.51 ± 0.82	4.43 ± 0.83	0.143
Knee stability	3.67 ± 1.26	3.67 ± 1.24	0.967
Satisfaction with the knee	4.68 ± 0.68	4.59 ± 0.82	0.049
Walking distance	3.13 ± 1.23	3.16 ± 1.22	0.706

### Results synthesis

At the 1-year follow-up, patients undergoing robotic-assisted TKA demonstrated superior functional outcomes compared with the conventional cohort (Table 2). Specifically, the VAS was lower in the robotic-assisted group (MD −0.75, 95% CI −0.94 to −0.56; *P* < 0.001). Similarly, all WOMAC domains favoured robotic-assisted TKA, including pain (MD −1.71, 95% CI −2.08 to −1.33; *P* < 0.001), stiffness (MD −0.74, 95% CI −0.94 to −0.53; *P* < 0.001) and function (MD −3.70, 95% CI −4.71 to −2.69; *P* < 0.001). Consequently, the overall WOMAC score was significantly lower in the robotic-assisted group (MD −6.14, 95% CI −7.47 to −4.81; *P* < 0.001). Patients in the CORI cohort also demonstrated superior Tegner scores (MD 0.33; 95% CI 0.17–0.50; *P* < 0.001). Furthermore, patients treated with the robotic-assisted technique reported greater satisfaction with the knee (MD −0.32; 95% CI −0.41 to −0.23; *P* < 0.001) and with the overall TKA outcome (MD −0.21; 95% CI −0.29 to −0.14; *P* < 0.001). Patients in the robotic-assisted cohort reported better performance in daily activities (MD −0.10; 95% CI −0.21 to −0.00; *P* = 0.048). The robotic-assisted group also showed a lower need for breaks during walking, corresponding to a lower score (MD −0.16; 95% CI −0.26 to −0.06; P = 0.002), less walking-distance limitation (MD −0.16; 95% CI −0.26 to −0.06; *P* = 0.001), greater perceived knee stability (MD 0.28; 95% CI 0.15 to 0.41; *P* < 0.001) and greater walking-distance capacity (MD −0.25; 95% CI −0.34 to −0.15; *P* < 0.001). No significant between-group difference was observed in the need for walking aids (MD −0.12; 95% CI −0.27 to +0.03; *P* = 0.126).

**Table 2:** Main results

Variable	Robotic-assisted (*N* = 383)	Conventional (*N* = 445)	MD	95% CI	*P*-value
Tegner	3.07 ± 1.24	2.74 ± 1.21	0.33	0.17 to 0.50	< 0.001
VAS (0–10)	1.17 ± 1.37	1.92 ± 1.65	−0.75	−0.94 to −0.56	< 0.001
WOMAC Pain	2.63 ± 2.64	4.33 ± 3.31	−1.71	−2.08 to −1.33	< 0.001
WOMAC Stiffness	1.00 ± 1.42	1.73 ± 1.76	−0.74	−0.94 to −0.53	< 0.001
WOMAC Function	10.23 ± 7.22	13.93 ± 8.80	−3.70	−4.71 to −2.69	< 0.001
WOMAC Total	13.86 ± 9.43	20.00 ± 11.61	−6.14	−7.47 to −4.81	< 0.001
Walking aids	1.69 ± 1.13	1.81 ± 1.17	−0.12	−0.27 to 0.03	0.126
Restricted daily activities	1.82 ± 0.76	1.92 ± 0.77	−0.1	−0.21 to −0.00	0.048
Need breaks when walking	1.88 ± 0.76	2.04 ± 0.78	−0.16	−0.26 to −0.06	0.002
Walking distance limitation	1.42 ± 0.72	1.58 ± 0.76	−0.16	−0.26 to −0.06	0.001
Knee stability	4.23 ± 0.95	3.95 ± 1.09	0.28	0.15 to 0.41	< 0.001
Satisfaction with the knee	1.31 ± 0.60	1.62 ± 0.78	−0.32	−0.41 to −0.23	< 0.001
Satisfaction with TKA outcome	1.20 ± 0.49	1.42 ± 0.68	−0.21	−0.29 to −0.14	< 0.001
Walking distance	1.47 ± 0.67	1.71 ± 0.77	−0.25	−0.34 to −0.15	< 0.001

## Discussion

Robotic-assisted TKA, performed within a structured four-dimensional personalised alignment workflow, is associated with significantly better patient-reported outcomes at the 1-year follow-up compared with conventional freehand TKA using an adjusted mechanical alignment. The differences were consistent across all evaluated domains and were observed in a large cohort enrolled within a highly standardised peri-operative environment, where both groups received the same implant and underwent the same rehabilitation protocol. The magnitude of the observed differences is clinically meaningful. The improvements in WOMAC, VAS pain, Tegner activity score and several items of the dedicated functional questionnaire, including walking distance, perceived knee stability, restriction in daily activities and need for walking breaks, reflect clinically relevant differences in patients’ daily lives rather than merely statistical effects of a large sample size.

Before interpreting these findings, the clinical context in which this study was conducted warrants particular attention, as it directly strengthens the validity of the comparison. The present study was conducted at the **, an EndoCert-certified centre for maximal arthroplasty care (Endoprothetikzentrum der Maximalversorgung, EPZmax). The institution has repeatedly received the highest quality rating in the AOK Hospital Navigator for hip and knee arthroplasty and performs approximately 2000 arthroplasty procedures annually. EndoCert is the official certification system of the German Society for Orthopaedics and Orthopaedic Surgery, developed to standardise and continuously monitor the quality of arthroplasty care in Germany [32]. The certification process requires strict adherence to pre-defined structural, organisational and clinical quality standards, including minimum annual surgical volumes, standardised peri-operative protocols, continuous outcome monitoring, complication tracking and regular annual external audits [32]. Surgeons operating within EndoCert-certified centres must demonstrate substantial experience in arthroplasty surgery and fulfil specific procedural volume requirements to obtain and maintain their certification as senior arthroplasty surgeons [32]. This context is not a minor detail. As both cohorts were treated within the same institutional environment by experienced arthroplasty surgeons, the observed functional differences are less likely to reflect differences in surgical expertise or institutional standards. Moreover, robotic assistance represented an established surgical workflow rather than a technology in its implementation phase. This is relevant because the initial learning curve predominantly affects operative efficiency, while surgical times stabilise with increasing robotic experience and may become comparable to established computer-assisted workflows [40, 41]. Instead, the differences may partly result from the way the surgery was planned and performed. Robotic-assisted TKA differs from conventional TKA not only by the use of a technological platform, but by integrating pre-resection soft-tissue evaluation, intra-operative adjustment of the component positioning, quantitative gap assessment and constrained execution of the final plan. The present findings therefore support the concept that the clinical impact of robotic-assisted TKA may derive from the combined effect of a more structured and data-informed surgical workflow rather than from the robotic device alone. The conventional group underwent a well-established and legitimate approach, with the femoral and tibial cuts targeting the mechanical axis and individual intra-operative adjustments made based on the surgeon’s clinical assessment [35, 36]. This is a sound technique, but it has inherent limitations: alignment decisions rely on anatomical landmarks and manual tactile feedback, soft-tissue balance is assessed qualitatively without numerical reference and there is no systematic framework that integrates planning across multiple planes simultaneously before any bone is resected [36, 42, 43]. The robotic workflow, by contrast, required a structured pre-operative morphotype assessment based on the CPAK classification, a personalised alignment plan adapted to individual bony anatomy, virtual soft-tissue gap simulation across the range of motion before any bone was cut, and an objective, reproducible balancing assessment using the KBS [6, 16, 29, 44]. These are not refinements of the same process; they represent a fundamentally different surgical logic.

The four-dimensional workflow applied in the robotic group draws from functional alignment principles, aiming to reconstruct constitutional knee anatomy within defined safe boundaries rather than targeting a fixed neutral mechanical axis for all patients [16, 45–47]. The CPAK-based morphotype classification allowed surgical targets to be adapted to individual anatomy at the planning stage, with explicit strategies defined for each category [34, 45]. This is particularly relevant for type C, where the degree of deformity makes it impossible to simultaneously restore native alignment and achieve soft-tissue balance without a systematic pre-operative strategy [34, 43, 46]. In conventional adjusted mechanical alignment, these patients are managed reactively, with soft-tissue releases added as needed intra-operatively [36, 48]. In the robotic workflow, the management strategy is anticipated, planned and executed within a framework that sets clear limits and defines an exit strategy before the patient enters the operating room [16, 20, 45].

The KBS played a specific and important role within this workflow [29]. Soft-tissue balance in total knee arthroplasty has historically been assessed by tactile perception or qualitative descriptors, concepts that are difficult to reproduce and even harder to teach. The KBS replaces this subjective assessment with a numerical classification based on the absolute mediolateral gap difference, measured separately in extension and flexion and grounded in the physiological behaviour of the native knee [29]. The rationale is straightforward: in extension, mediolateral symmetry is required for coronal stability during stance and any medial opening greater than 2 mm is not tolerated; in flexion, a controlled lateral laxity between 2 and 4 mm is physiological and reflects the natural external rotation mechanism of the tibia during flexion, whereas medial laxity or excessive lateral opening are pathological [29]. The capacity to detect, classify and correct these imbalances before committing to bone cuts, rather than discovering them after resection, is a structural advantage of the robotic workflow that we believe directly contributed to the functional outcomes observed [48, 49].

A further element worth discussing is the role of the tibial bone cut as a functional soft-tissue release event, a concept that informed the interpretation of intra-operative gap measurements in the robotic group. In varus knees with low MPTA, the proximal tibial resection releases fibres of the deep medial collateral ligament and the proximal superficial medial collateral ligament, functionally reducing medial tightness [50, 51]. In knees with an elevated MPTA, the same cut may partially release anterolateral capsular structures, including the anterolateral ligament complex, affecting lateral tension [52, 53]. Recognising this effect and re-evaluating balance after tibial resection, rather than treating pre-cut measurements as definitive, allowed more accurate final balancing and reduced the need for additional soft-tissue releases [54, 55]. This dynamic interpretation of intra-operative data is possible only when the surgeon has a structured framework and objective measurements to work with, which, in turn, points to the workflow rather than the robot as the key variable.

Several limitations of this study should be acknowledged. The quasi-randomised design, while based on an objective computer-generated allocation mechanism, does not provide the same level of bias control as true randomisation, and residual confounding cannot be fully excluded. An additional source of potential confounding is that the surgeon and surgical technique were not independent, as two surgeons routinely performed robotic-assisted TKA and two performed conventional TKA. Therefore, surgeon-specific effects cannot be fully distinguished from those associated with the surgical technique and workflow. The study was conducted at a single specialised centre, and results may not be directly generalisable to settings with lower surgical volumes or less experienced teams. The 1-year follow-up, while sufficient to capture early functional recovery and patient satisfaction, does not address long-term implant survival or the durability of the observed functional differences.

## Conclusions

Robotic-assisted TKA was associated with superior functional and patient-reported outcomes at the 1-year follow-up. The clinical benefit observed is likely not attributable to robotic precision alone, but reflects the structured, personalised intra-operative workflow that robotic assistance enables.

## Data Availability

Data are available upon reasonable request to the corresponding author.

## References

[1] Stubnya BG, Schulz M, Vancsa S, Szilagyi GS, Szatmari A, Bejek Z (2025) Global trends in joint arthroplasty: a systematic review and future projections. J Clin Med. 10.3390/jcm14228214 PMC12653837 41303249

[2] Migliorini F, Maffulli N, Schafer L, Simeone F, Bell A, Hofmann UK (2024) Minimal clinically important difference (MCID), substantial clinical benefit (SCB), and patient-acceptable symptom state (PASS) in patients who have undergone total knee arthroplasty: a systematic review. Knee Surg Relat Res 36(1):3. 10.1186/s43019-024-00210-z PMC10782530 38212863

[3] Xu Y, Wang W (2025) A meta-analysis of the therapeutic effect of total knee replacement after knee arthroscopic surgery. J Orthop Traumatol 26(1):31. 10.1186/s10195-025-00848-9 PMC12098253 40402175

[4] DeFrance MJ, Scuderi GR (2023) Are 20% of patients actually dissatisfied following total knee arthroplasty? A systematic review of the literature. J Arthroplasty 38(3):594–599. 10.1016/j.arth.2022.10.011 36252743

[5] Nakano N, Shoman H, Olavarria F, Matsumoto T, Kuroda R, Khanduja V (2020) Why are patients dissatisfied following a total knee replacement? A systematic review. Int Orthop 44(10):1971–2007. 10.1007/s00264-020-04607-9 PMC7584563 32642827

[6] Migliorini F, Pilone M, Schafer L, Simeone F, Bell A, Maffulli N (2024) Functional alignment in robotic-assisted total knee arthroplasty: a systematic review. Arch Orthop Trauma Surg 144(4):1741–1749. 10.1007/s00402-023-05195-0 38337093

[7] Lustig S, Sappey-Marinier E, Fary C, Servien E, Parratte S, Batailler C (2021) Personalized alignment in total knee arthroplasty: current concepts. SICOT J 7:19. 10.1051/sicotj/2021021 PMC8019550 33812467

[8] Bellemans J, Colyn W, Vandenneucker H, Victor J (2012) The Chitranjan Ranawat award: is neutral mechanical alignment normal for all patients? The concept of constitutional varus. Clin Orthop Relat Res 470(1):45–53. 10.1007/s11999-011-1936-5 PMC3237976 21656315

[9] Migliorini F, Maffulli N, Pilone M, Schafer L, Ullmann D, Huber T, Rath B (2025) Mechanical versus kinematic alignment for total knee arthroplasty: a meta-analysis. Arch Orthop Trauma Surg 145(1):212. 10.1007/s00402-025-05835-7 40133714

[10] Roussot MA, Vles GF, Oussedik S (2020) Clinical outcomes of kinematic alignment versus mechanical alignment in total knee arthroplasty: a systematic review. EFORT Open Rev 5(8):486–497. 10.1302/2058-5241.5.190093 PMC7484715 32953134

[11] Shih CM, Chen KH, Jiang FC, Hsu CE, Wang CC, Wang SP (2025) Short-term outcomes of robotic versus conventional unicompartmental knee arthroplasty: evidence from a national database. J Orthop Traumatol 26(1):74. 10.1186/s10195-025-00896-1 PMC12672964 41329432

[12] Yang C, Ji B, Li G, Zhang X, Xu B, Maimaitiming A, Cao L (2025) Weight-based tranexamic acid lowers the risk of postoperative blood loss and transfusion requirements compared with fixed-dose regimen in revision knee arthroplasty: a comparative study. J Orthop Traumatol 26(1):26. 10.1186/s10195-025-00844-z PMC12048371 40316735

[13] MacDessi SJ, Griffiths-Jones W, Chen DB, Griffiths-Jones S, Wood JA, Diwan AD, Harris IA (2020) Restoring the constitutional alignment with a restrictive kinematic protocol improves quantitative soft-tissue balance in total knee arthroplasty: a randomized controlled trial. Bone Joint J 102-B(1):117–124. 10.1302/0301-620X.102B1.BJJ-2019-0674.R2 PMC6974544 31888372

[14] Moser LB, Hess S, Amsler F, Behrend H, Hirschmann MT (2019) Native non-osteoarthritic knees have a highly variable coronal alignment: a systematic review. Knee Surg Sports Traumatol Arthrosc 27(5):1359–1367. 10.1007/s00167-019-05417-2 30848310

[15] von Eisenhart-Rothe R, Lustig S, Graichen H, Koch PP, Becker R, Mullaji A, Hirschmann MT (2022) A safe transition to a more personalized alignment in total knee arthroplasty: the importance of a “safe zone” concept. Knee Surg Sports Traumatol Arthrosc 30(2):365–367. 10.1007/s00167-021-06844-w 35048141

[16] Migliorini F, Schafer L, Schneider J, Nobili AM, Kammer D, Bell A (2026) Improved accuracy of functional alignment restoration with robotic-assisted total knee arthroplasty: a quasi-randomized controlled trial. Orthopadie (Heidelb) 55(2):132–139. 10.1007/s00132-025-04711-x PMC12881118 40952461

[17] Pintore A, Notarfrancesco D, Zara A, Oliva F, Migliorini F, Maffulli N (2025) Kinematic alignment in total knee arthroplasty restores the native anatomy, with a joint line parallel to the ground on a standing view. J Orthop 69:142–149. 10.1016/j.jor.2025.05.051 PMC12167787 40529903

[18] Aflatooni JO, Wininger AE, Park KJ, Incavo SJ (2023) Alignment options and robotics in total knee arthroplasty. Front Surg 10:1106608. 10.3389/fsurg.2023.1106608 PMC9947398 36843989

[19] Migliorini F, Maffulli N, Schäfer L, Schneider J, Nobili AM, Kämmer D, Michalak M, Bell A (2023) Robotic-assisted total knee arthroplasty in clinical practice: protocol for a randomised controlled trial. J Orthop Surg Res 18(1):623. 10.1186/s13018-023-04101-z. PMID: 37626412; PMCID: PMC10464371. PMC10464371 37626412

[20] Wan X, Su Q, Wang D, Yuan M, Lai Y, Xu H, Zhou Z (2021) Robotic arm-assisted total knee arthroplasty improves preoperative planning and intraoperative decision-making. J Orthop Surg Res 16(1):670. 10.1186/s13018-021-02815-6 PMC8591833 34781977

[21] St Mart JP, Goh EL (2021) The current state of robotics in total knee arthroplasty. EFORT Open Rev 6(4):270–279. 10.1302/2058-5241.6.200052 PMC8142057 34040804

[22] Hiranaka T (2024) Current concept: personalized alignment total knee arthroplasty as a contrast to classical mechanical alignment total knee arthroplasty. Arthroplasty (Heidelb) 6(1):23. 10.1186/s42836-024-00246-2 PMC11071279 38705976

[23] Fan Y, Chen S, Jiang L, Chen Z, Wu M, Wang C (2026) Effects of anterior cruciate ligament resection and total knee arthroplasty on lower limb alignment and tibiofemoral rotation. J Orthop Traumatol. 10.1186/s10195-026-00942-6 PMC13490352 42262630

[24] Ruangsomboon P, Ruangsomboon O, Pornrattanamaneewong C, Narkbunnam R, Chareancholvanich K (2023) Clinical and radiological outcomes of robotic-assisted versus conventional total knee arthroplasty: a systematic review and meta-analysis of randomized controlled trials. Acta Orthop 94:60–79. 10.2340/17453674.2023.9411 PMC9941983 36805771

[25] Mostafa O, Malik M, Qayum K, Ishaq U, Khan AM, Wasim AS, Alsoud Z, Quraishi S (2025) Robotic-assisted versus conventional total knee arthroplasty: a systematic review and meta-analysis of alignment accuracy and clinical outcomes. Ann Med Surg (Lond) 87(2):867–879. 10.1097/MS9.0000000000002919 PMC11918693 40110313

[26] Alrajeb R, Zarti M, Shuia Z, Alzobi O, Ahmed G, Elmhiregh A (2024) Robotic-assisted versus conventional total knee arthroplasty: a systematic review and meta-analysis of randomized controlled trials. Eur J Orthop Surg Traumatol 34(3):1333–1343. 10.1007/s00590-023-03798-2 PMC10980635 38133653

[27] Morcos MW, Uhuebor D, Vendittoli PA (2023) Overview of the different personalized total knee arthroplasty with robotic assistance, how choosing? Front Surg 10:1120908. 10.3389/fsurg.2023.1120908 PMC10020354 36936647

[28] Holzapfel DE, Kappenschneider T, Holzapfel S, Schuster MF, Michalk K, Auer P, Schwarz T (2025) Intensive care needs after hip and knee replacement: understanding risk profiles for severe postoperative complications. J Orthop Traumatol 26(1):42. 10.1186/s10195-025-00862-x PMC12229386 40608156

[29] Bell A, Schafer L, Nobili A, Kammer D, Schneider J, Bell M, Migliorini F (2026) Intraoperative balance in total knee arthroplasty : development and internal validation of the Knee Balancing Score. Orthopadie (Heidelb). 10.1007/s00132-026-04810-3 41915183

[30] Hopewell S, Chan AW, Collins GS, Hrobjartsson A, Moher D, Schulz KF, Tunn R, Aggarwal R, Berkwits M, Berlin JA, Bhandari N, Butcher NJ, Campbell MK, Chidebe RCW, Elbourne D, Farmer A, Fergusson DA, Golub RM, Goodman SN, Hoffmann TC, Ioannidis JPA, Kahan BC, Knowles RL, Lamb SE, Lewis S, Loder E, Offringa M, Ravaud P, Richards DP, Rockhold FW, Schriger DL, Siegfried NL, Staniszewska S, Taylor RS, Thabane T, Torgerson D, Vohra S, White IR, Boutron I (2025) CONSORT 2025 statement: updated guideline for reporting randomised trials. BMJ 389:e081123. 10.1136/bmj-2024-081123 PMC11995449 40228833

[31] Migliorini F, Randelli F, Di Martino A, Rivera F (2025) Writing for JOOT: raising standards in clinical research and evidence synthesis. J Orthop Traumatol. 10.1186/s10195-025-00868-5 PMC12321706 40760235

[32] Haas H, Mittelmeier W (2014) Implementation of the EndoCert system for certification of arthroplasty centers. Exp Pilot Phase Orthopade 43(6):534–540. 10.1007/s00132-014-2294-2 24928271

[33] Kellgren JH, Lawrence JS (1957) Radiological assessment of osteo-arthrosis. Ann Rheum Dis 16(4):494–502. 10.1136/ard.16.4.494 PMC1006995 13498604

[34] MacDessi SJ, Griffiths-Jones W, Harris IA, Bellemans J, Chen DB (2021) Coronal plane alignment of the knee (CPAK) classification. Bone Joint J 103-B(2):329–337. 10.1302/0301-620X.103B2.BJJ-2020-1050.R1 PMC7954147 33517740

[35] Weber P, Gollwitzer H (2022) Arthroplasty of the knee: current techniques for implant alignment. Z Orthop Unfall 160(2):149–159. 10.1055/a-1304-3854 PMC8967431 33647995

[36] Daines BK, Dennis DA (2014) Gap balancing vs. measured resection technique in total knee arthroplasty. Clin Orthop Surg 6(1):1–8. 10.4055/cios.2014.6.1.1 PMC3942594 24605183

[37] Tegner Y, Lysholm J (1985) Rating systems in the evaluation of knee ligament injuries. Clin Orthop Relat Res 198:43–49 4028566

[38] Butler PV (1997) Linear analogue self-assessment and procrustean measurement: a critical review of visual analogue scaling in pain assessment. J Clin Psychol Med Settings 4(1):111–129. 10.1023/A:1026240322240

[39] Bellamy N, Buchanan WW, Goldsmith CH, Campbell J, Stitt LW (1988) Validation study of WOMAC: a health status instrument for measuring clinically important patient relevant outcomes to antirheumatic drug therapy in patients with osteoarthritis of the hip or knee. J Rheumatol 15(12):1833–1840 3068365

[40] Haslhofer DJ, Anelli-Monti V, Hausbrandt P, Kammerlander C, Klasan A (2024) No difference in surgical time and total theatre time between robotically assisted and computer assisted total knee arthroplasty. J Orthop Traumatol 25(1):52. 10.1186/s10195-024-00798-8 PMC11549068 39516337

[41] Migliorini F, Schafer L, Lucenti L, Schneider J, Nobili AM, Kammer D, Maffulli N, Bell A (2026) Learning curve for robotic-assisted total knee arthroplasty. J Orthop Surg Res. 10.1186/s13018-026-06835-y PMC13130575 41943084

[42] Gordon AC, Conditt MA, Verstraete MA (2021) Achieving a balanced knee in robotic TKA. Sensors (Basel). 10.3390/s21020535 PMC7828562 33451019

[43] Clark G, Steer R, Wood D (2023) Functional alignment achieves a more balanced total knee arthroplasty than either mechanical alignment or kinematic alignment prior to soft tissue releases. Knee Surg Sports Traumatol Arthrosc 31(4):1420–1426. 10.1007/s00167-022-07156-3 PMC10050049 36116071

[44] Migliorini F, Schafer L, Schneider J, Nobili AM, Kammer D, Maffulli N, Bell A (2026) Coronal plane alignment of the knee (CPAK) phenotypes and their relationship with gap patterns in robotic-assisted total knee arthroplasty: a clinical trial. Orthopadie (Heidelb). 10.1007/s00132-026-04797-x PMC13525067 41876880

[45] MacDessi SJ (2021) Restricted kinematic alignment in total knee arthroplasty: scientific exploration involving detailed planning, precise execution, and knowledge of when to abort. Arthroplast Today 10:24–26. 10.1016/j.artd.2021.05.024 PMC8267482 34277907

[46] Shatrov J, Battelier C, Sappey-Marinier E, Gunst S, Servien E, Lustig S (2022) Functional Alignment Philosophy in Total Knee Arthroplasty - Rationale and technique for the varus morphotype using a CT based robotic platform and individualized planning. SICOT J 8:11. 10.1051/sicotj/2022010 PMC8973302 35363136

[47] Migliorini F, Pilone M, Schafer L, Vaishya R, Moretti G, Memminger M, Maffulli N (2026) Alignment strategy does not confer clinically meaningful advantages in medial pivot total knee arthroplasty: a systematic review and meta-analysis. J Orthop Surg Res. 10.1186/s13018-026-06668-9 PMC13063706 41877255

[48] Matsumoto T, Nakano N, Tsubosaka M, Muratsu H (2024) Soft tissue balance in total knee arthroplasty: clinical value of intra-operative measurement. J Joint Surg Res 2(3):85–93. 10.1016/j.jjoisr.2024.05.001

[49] Migliorini F, Eschweiler J, Mansy YE, Quack V, Schenker H, Tingart M, Driessen A (2020) Gap balancing versus measured resection for primary total knee arthroplasty: a meta-analysis study. Arch Orthop Trauma Surg 140(9):1245–1253. 10.1007/s00402-020-03478-4 32409905

[50] Kim MW, Koh IJ, Kim JH, Jung JJ, In Y (2015) Efficacy and safety of a novel three-step medial release technique in varus total knee arthroplasty. J Arthroplasty 30(9):1542–1547. 10.1016/j.arth.2015.03.037 25922122

[51] Aberman Z, Germano J, Scuderi GR (2024) Intraoperative medial instability during total knee arthroplasty. Orthop Clin North Am 55(1):61–71. 10.1016/j.ocl.2023.06.005 37980104

[52] Rossi R, Rosso F, Cottino U, Dettoni F, Bonasia DE, Bruzzone M (2014) Total knee arthroplasty in the valgus knee. Int Orthop 38(2):273–283. 10.1007/s00264-013-2227-4 PMC3923939 24366186

[53] Kraeutler MJ, Welton KL, Chahla J, LaPrade RF, McCarty EC (2018) Current concepts of the anterolateral ligament of the knee: anatomy, biomechanics, and reconstruction. Am J Sports Med 46(5):1235–1242. 10.1177/0363546517701920 28426251

[54] Orsi AD, Wakelin EA, Plaskos C, Gupta S, Sullivan JA (2022) Predictive gap-balancing reduces the extent of soft-tissue adjustment required after bony resection in robot-assisted total knee arthroplasty-A comparison with simulated measured resection. Arthroplast Today 16:1–8. 10.1016/j.artd.2022.03.025 PMC9126743 35620585

[55] Shalhoub S, Lawrence JM, Keggi JM, Randall AL, DeClaire JH, Plaskos C (2019) Imageless, robotic-assisted total knee arthroplasty combined with a robotic tensioning system can help predict and achieve accurate postoperative ligament balance. Arthroplast Today 5(3):334–340. 10.1016/j.artd.2019.07.003 PMC6728592 31516978

